# Supplementary material for: Inhibition of NMDA receptors through a membrane-to-channel path
Source: Nat Commun. 2022 Jul 15;13:4114. doi: 10.1038/s41467-022-31817-z (PMC9287434; doi:10.1038/s41467-022-31817-z)
Supplement: Supplementary file 1 — Supplementary Information [file 41467_2022_31817_MOESM1_ESM.pdf]

# SUPPLEMENTARY INFORMATION
# Inhibition of NMDA Receptors Through a Membrane-to-Channel Path

Madeleine R. Wilcox, Aparna Nigam, Nathan G. Glasgow, Chamali Narangoda, Matthew B. Phillips, Dhilon S. Patel, Samaneh Mesbahi-Vasey, Andreea L. Turcu, Santiago Vázquez, Maria G. Kurnikova, and Jon W. Johnson

## SUPPLEMENTARY METHODS

**Chemical synthesis.** Commercially available reagents and solvents were used without further purification unless stated otherwise. Melting points were determined in open capillary tubes with an MFB 595010M Gallenkamp. The structures of DMM and TMM were confirmed by elemental analysis and accurate mass measurement, IR, $^{1}$H NMR (Supplementary Figs. 4 and 8), and $^{13}$C NMR (Supplementary Figs. 5 and 9).

400 MHz $^{1}$H and 100.6 MHz $^{13}$C NMR spectra were recorded on a Varian Mercury spectrometer. The chemical shifts are reported in ppm (δ scale) relative to internal tetramethylsilane, and coupling constants are reported in Hertz (Hz). Assignments given for the NMR spectra of DMM and TMM were carried out based on COSY $^{1}$H/$^{1}$H (standard procedures; Supplementary Figs. 6 and 10), and COSY $^{1}$H/$^{13}$C (gHSQC) experiments (Supplementary Figs. 7 and 11). IR spectra were run on a PerkinElmer Spectrum TWO. Absorption values are expressed as wave-numbers (cm$^{-1}$); only significant absorption bands are given. High-resolution mass spectrometry (HRMS) analyses were performed with an LC/MSD TOF Agilent Technologies spectrometer. The elemental analyses were carried out in a Flash 1112 series thermofinnigan elemental microanalyzator (A5) to determine C, H, and N.

The purity of the sample was evaluated using an HPLC Agilent 1260 Infinity II LC/MSD coupled to a photodiode array and mass spectrometer. The samples were injected in an Agilent Poroshell 120 EC-C18, 2.7 µm, 50 mm×4.6 mm column at 40°C and 0.6 mL/min flow. The mobile phase was a mixture of A = water with 0.05% formic acid and B = acetonitrile with 0.05% formic acid. To detect memantine, DMM and TMM, sim and scan modes were used. The procedure followed for sim mode was from 95% A−5% B to 100% B in 3 min, 100% B 3 min, from 100% B to 95% A−5% B in 1 min, 95% A−5% B 3 min, 5 µL injection of 0.1 mg/mL; and for scan mode from 95% A−5% B to 80% A-20% B in 4 min, from 80% A-20% B to 50% A-50% B in 9 min, from 50% A-50% B to 95% A−5% B in 1 min, 1 µL injection of 0.5 mg/mL for scan mode. The limit of detection was determined by analyzing known concentrations of memantine and DMM using sim mode.

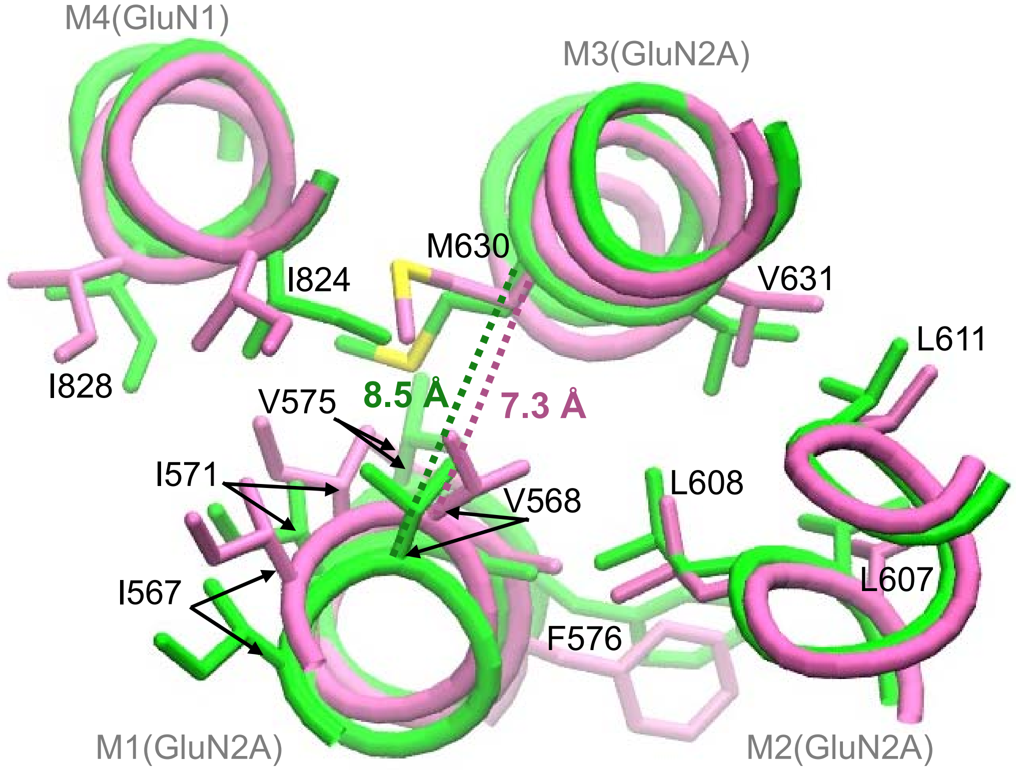


**Supplementary Figure 1. A comparison of the fenestration in closed and open NMDAR models.** View from the extracellular solution of a portion of the fenestration in closed (mauve) and open (green) NMDAR Model 2. The figure shows representative structures aligned using the backbone heavy atoms of the residues that line the membrane-to-channel path in the open channel (shown in stick representation and labeled; sulfur, yellow). The opening of the channel results in a slight increase of the distance between M1 and M3 helices of the GluN2A subunit and relative repositing of the side chains of M1 residues near the path. The labeled distance between the Cα atoms of GluN2A(V568) of M1 and GluN2A(M630) of M3 increases by 1.2 Å.

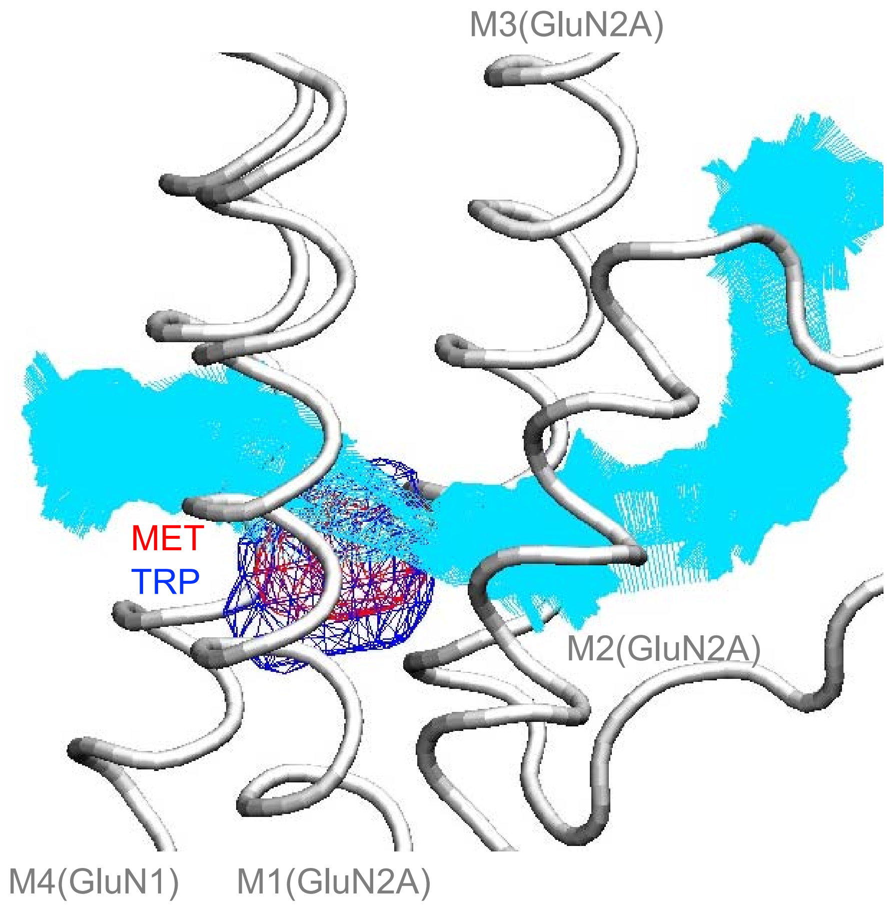


**Supplementary Figure 2. Simulated positions of methionine and tryptophan side chains at GluN2A(630).** The side chain density of the wildtype (WT) methionine residue (red mesh) and mutant tryptophan residue (blue mesh) at position GluN2A(630). Densities are averages over 200 ns of equilibrium MD simulations using NMDAR Model 2 performed in the absence of channel blocker. The trajectory of memantine obtained by steered MD simulations is shown in cyan.

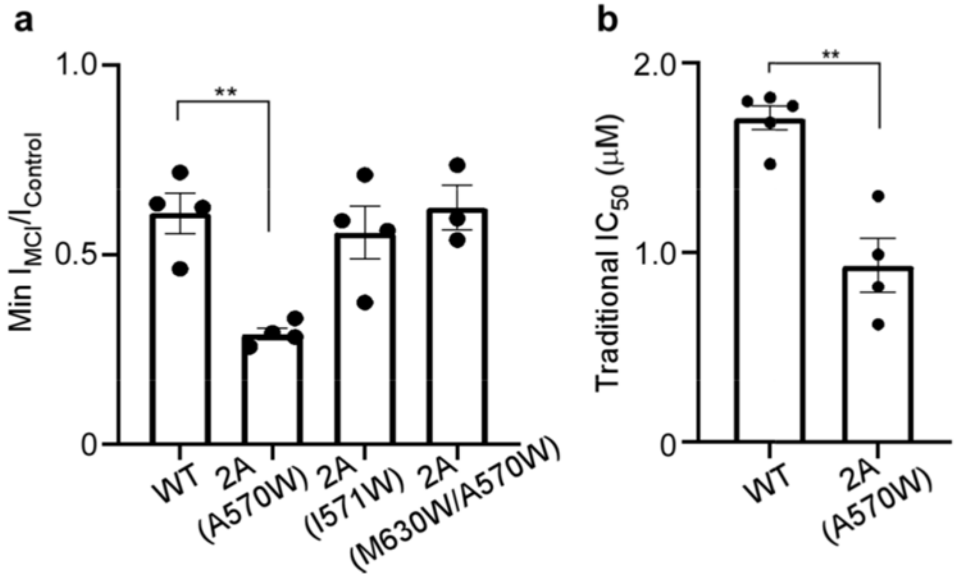


**Supplementary Figure 3. Mutation of additional fenestration-lining residues. a** Min $I_{MCI}/I_{Control}$ values measured with 100 μM memantine at pH 7.2 for WT, GluN1/2A(A570W), GluN1/2A(I571W), and GluN1/2A(M630W/A570W) receptors. Cells transfected to express GluN1/2A(M630W)(I571W) receptors also were examined, but showed no glutamate-activated current in 3 of 3 cells. The Min $I_{MCI}/I_{Control}$ values were: WT receptors, 0.610 ± 0.053, n = 4; GluN1/2A(A570W) receptors, 0.291 ± 0.016, n = 4; GluN1/2A(I571W) receptors, 0.560 ± 0.070, n = 4; GluN1/2A(M630W/A570W), 0.625 ± 0.059, n = 3. Values compared with one-way ANOVA ($F(3,11) = 8.87$, $p = 0.0028$) and Tukey post-hoc test (WT vs GluN1/2A(A570W), $p = 0.0029$; WT vs GluN1/2A(I571W), $p = 0.83$; WT vs GluN1/2A(M630W/A570W), $p = 0.996$). **b** The traditional memantine $IC_{50}$ of WT receptors (1.71 ± 0.06 μM, n = 5) and GluN1/2A(A570W) receptors (0.933 ± 0.144 μM, n = 4) were compared with a two-tailed t-test ($t = 5.33$, df = 7, $p = 0.0011$). Mutation of GluN2A(A570) to tryptophan affected traditional memantine $IC_{50}$ as well as memantine MCI. n is the number of biologically independent cells. In **a**, **b**, mean ± SEM is plotted.

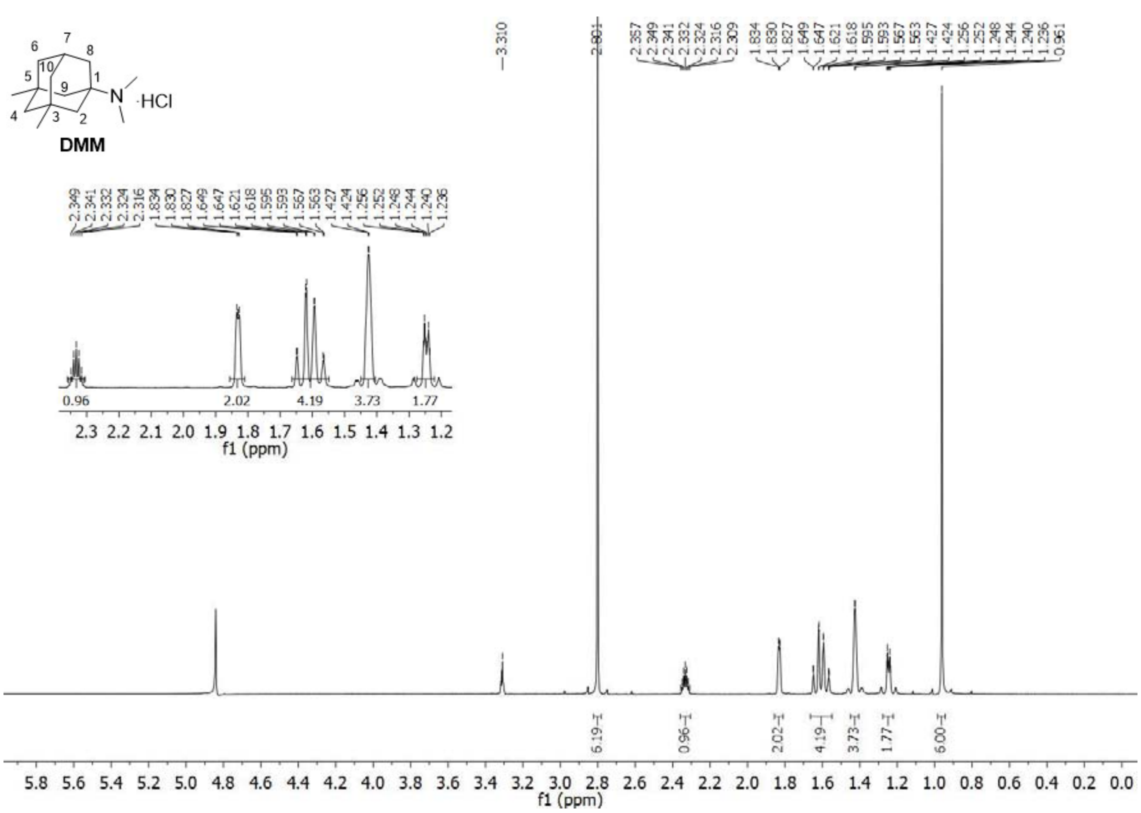


**Supplementary Figure 4. $^{1}$H NMR (400 MHz, $CD_3OD$) spectra for N,N,3,5-tetramethyladamantan-1-amine hydrochloride (DMM).**

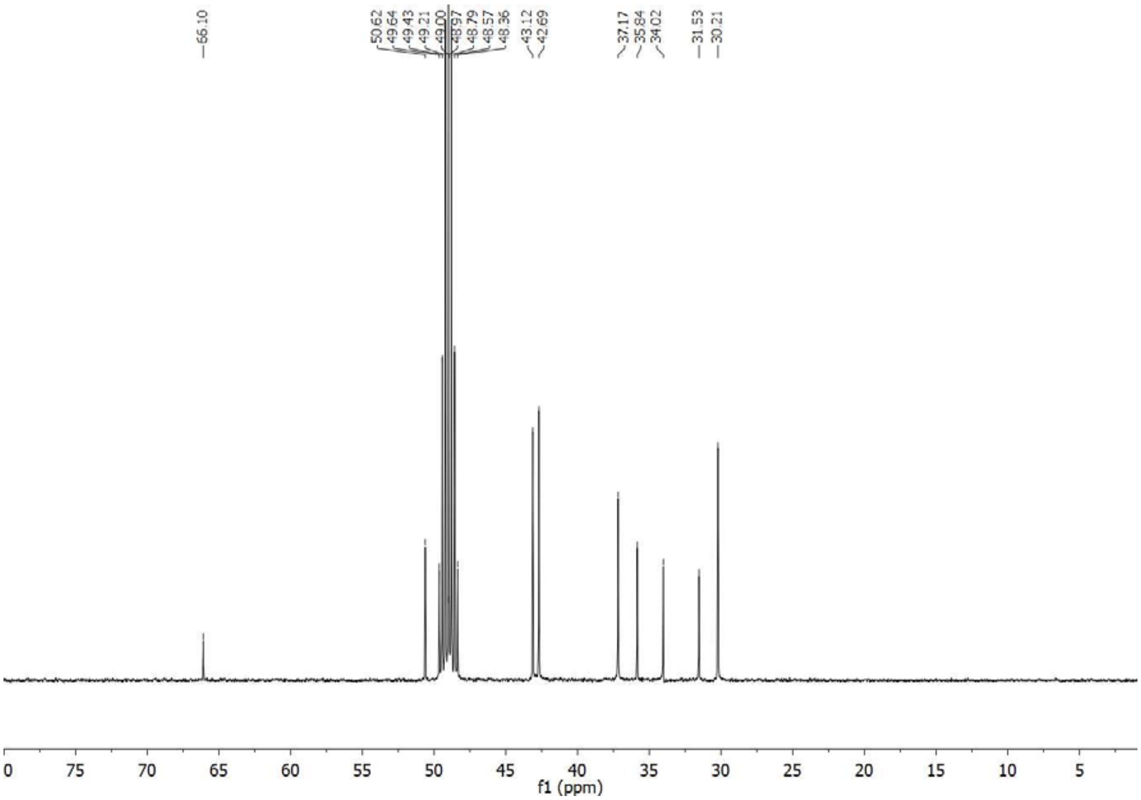


**Supplementary Figure 5. $^{13}$C-NMR (100.6 MHz, $CD_3OD$) spectra for DMM.**

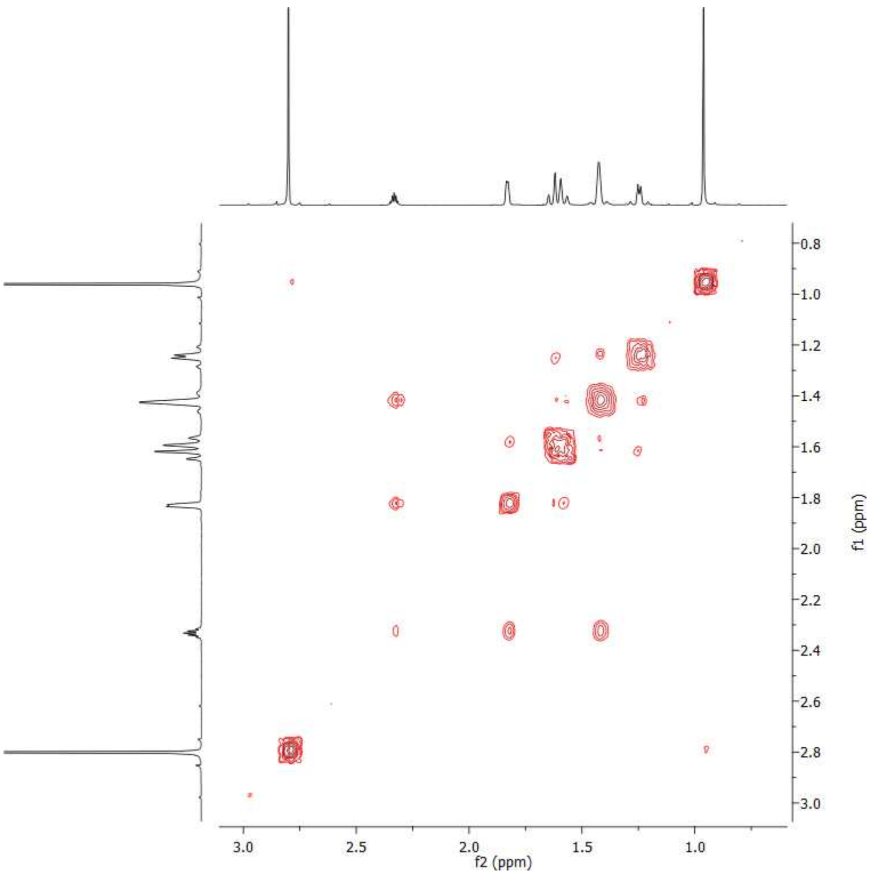


**Supplementary Figure 6. COSY NMR ($CD_3OD$) spectra for DMM.**

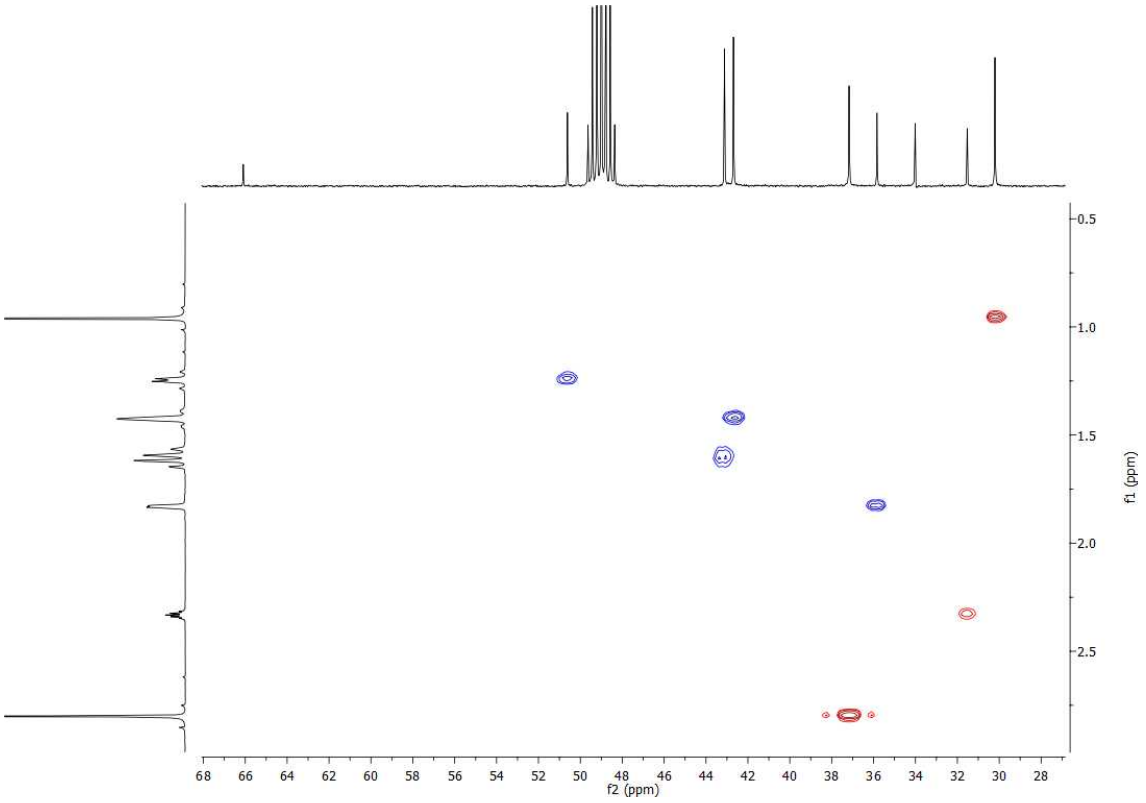


**Supplementary Figure 7. HSQC NMR ($CD_3OD$) spectra for DMM.**

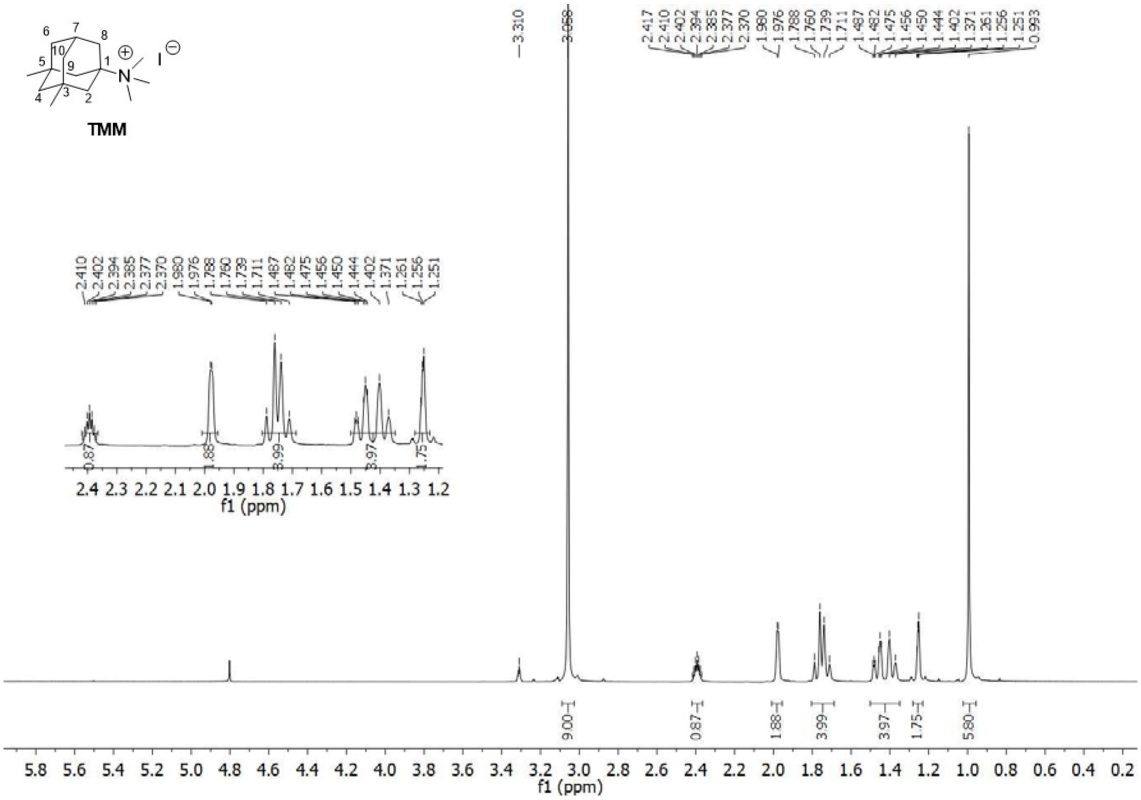


**Supplementary Figure 8. $^1$H NMR (400 MHz, $CD_3OD$) spectra for *N,N,N,*3,5-pentamethyladamantan-1-ammonium iodide (TMM).**

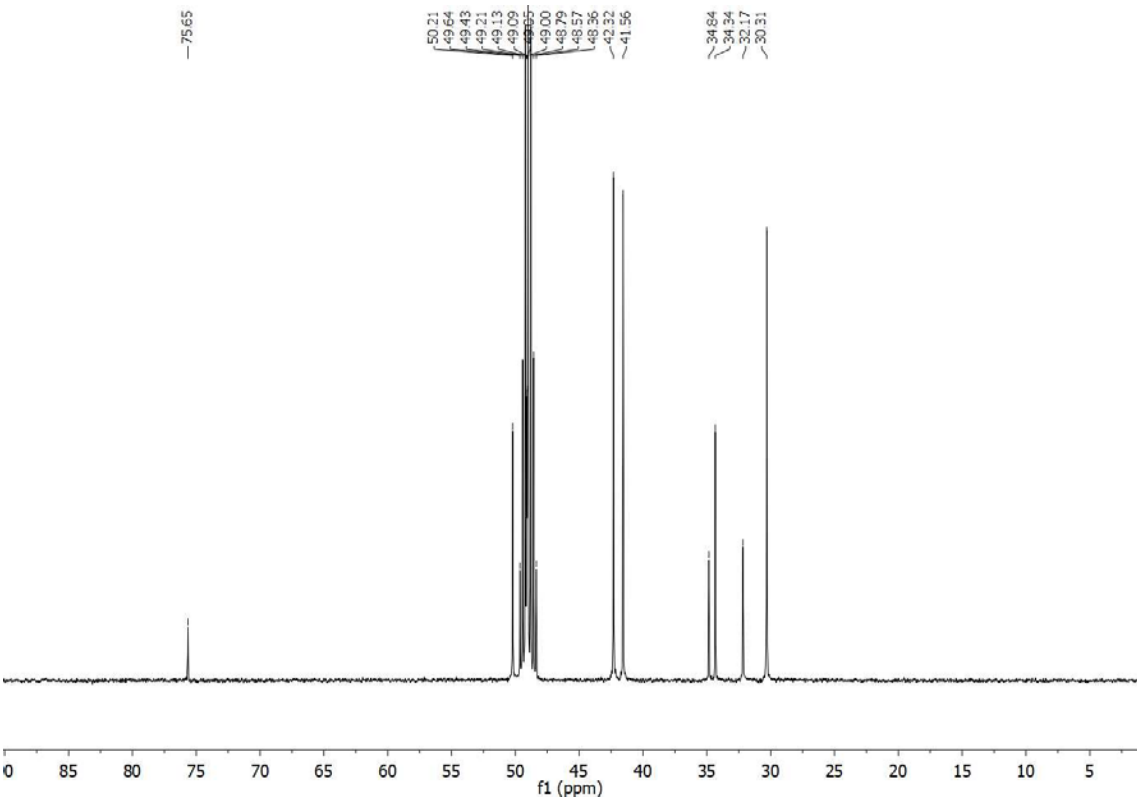


**Supplementary Figure 9. $^{13}$C-NMR (100.6 MHz, $CD_3OD$) spectra for TMM.**

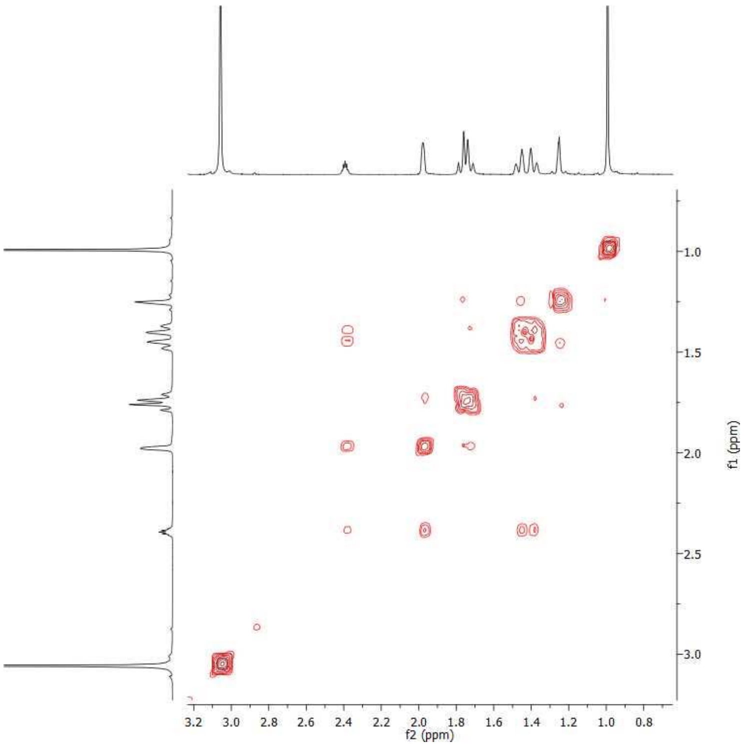


**Supplementary Figure 10. COSY NMR ($CD_3OD$) spectra for TMM.**

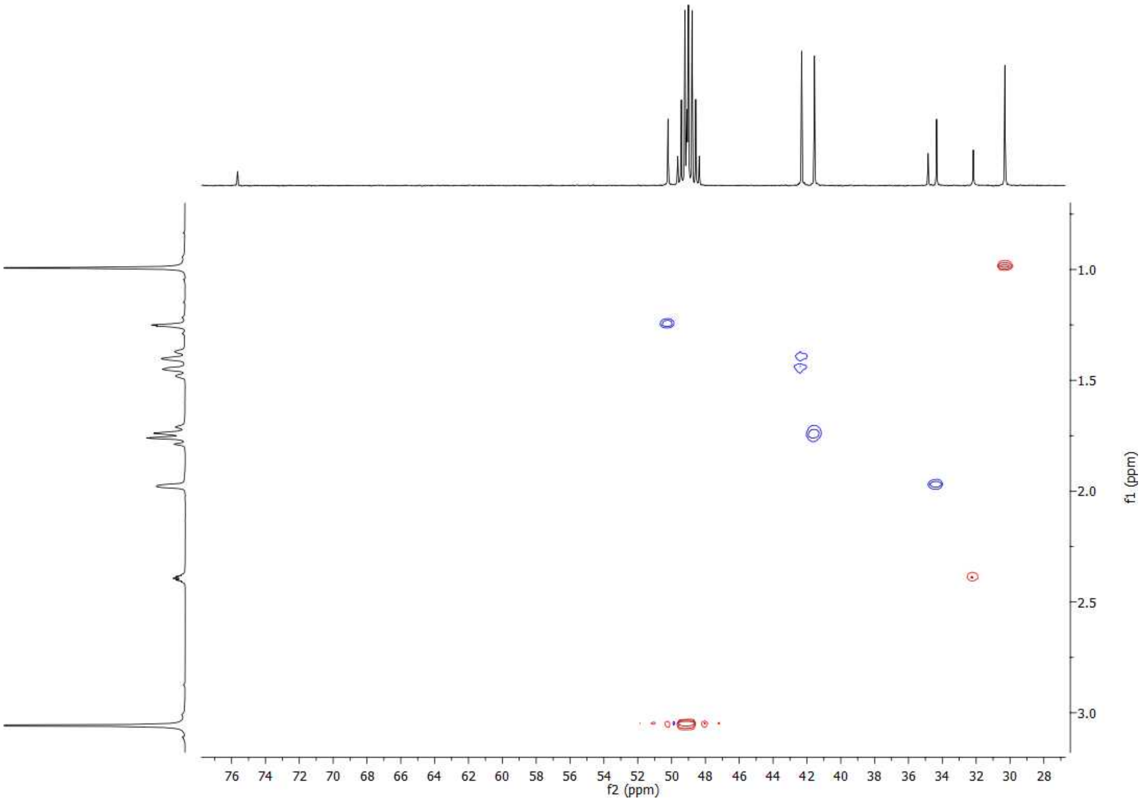


**Supplementary Figure 11. HSQC NMR ($CD_3OD$) spectra for TMM.**
