# Supplementary material for: Inhibition of NMDA receptors through a membrane-to-channel path
Source: Nat Commun. 2022 Jul 15;13:4114. doi: 10.1038/s41467-022-31817-z (PMC9287434; doi:10.1038/s41467-022-31817-z)
Supplement: Supplementary file 3 — Description of Additional Supplmentary Files [file 41467_2022_31817_MOESM3_ESM.docx]

**Description of Additional Supplementary Files**

File Name: Supplementary Movie 1

Description: Ion permeation in the open state NMDAR Model 2. Visualization of permeation of a potassium ion (red sphere) through the external channel gate. The M3 (silver) and M2 (green) helices of the GluN1 and GluN2A subunits are shown.
