# Supplementary material for: Inhibition of NMDA receptors through a membrane-to-channel path
Source: Nat Commun. 2022 Jul 15;13:4114. doi: 10.1038/s41467-022-31817-z (PMC9287434; doi:10.1038/s41467-022-31817-z)
Supplement: Supplementary file 5 — Reporting Summary [file 41467_2022_31817_MOESM5_ESM.pdf]

nature portfolio

Corresponding author(s): Jon W. Johnson

Last updated by author(s): 24 June 2022

Reporting Summary

Nature Portfolio wishes to improve the reproducibility of the work that we publish. This form provides structure for consistency and transparency in reporting. For further information on Nature Portfolio policies, see our Editorial Policies and the Editorial Policy Checklist.

Statistics

For all statistical analyses, confirm that the following items are present in the figure legend, table legend, main text, or Methods section.

n/a	Confirmed	
☐	☒	The exact sample size (*n*) for each experimental group/condition, given as a discrete number and unit of measurement
☐	☒	A statement on whether measurements were taken from distinct samples or whether the same sample was measured repeatedly
☐	☒	The statistical test(s) used AND whether they are one- or two-sided<br>*Only common tests should be described solely by name; describe more complex techniques in the Methods section.*
☒	☐	A description of all covariates tested
☐	☒	A description of any assumptions or corrections, such as tests of normality and adjustment for multiple comparisons
☐	☒	A full description of the statistical parameters including central tendency (e.g. means) or other basic estimates (e.g. regression coefficient) AND variation (e.g. standard deviation) or associated estimates of uncertainty (e.g. confidence intervals)
☐	☒	For null hypothesis testing, the test statistic (e.g. *F*, *t*, *r*) with confidence intervals, effect sizes, degrees of freedom and *P* value noted<br>*Give P values as exact values whenever suitable.*
☒	☐	For Bayesian analysis, information on the choice of priors and Markov chain Monte Carlo settings
☒	☐	For hierarchical and complex designs, identification of the appropriate level for tests and full reporting of outcomes
☒	☐	Estimates of effect sizes (e.g. Cohen's *d*, Pearson's *r*), indicating how they were calculated

Our web collection on statistics for biologists contains articles on many of the points above.

Software and code

Policy information about availability of computer code

Data collection	AMBER18 for molecular modeling<br>AutoDock Vina 1.2.0 for molecular docking<br>CHARMM-GUI 3.2 for molecular modeling<br>pClamp 10 for electrophysiological data acquisition
Data analysis	AMBER18 for analysis of molecular models<br>GraphPad Prism 7 for data analysis and presentation<br>HOLE v2.2 to identify paths through molecular model<br>Origin 16 for data analysis and presentation<br>pClamp 10 for electrophysiological data analysis<br>VMD 1.9.4 for molecular model visualization and analysis

For manuscripts utilizing custom algorithms or software that are central to the research but not yet described in published literature, software must be made available to editors and reviewers. We strongly encourage code deposition in a community repository (e.g. GitHub). See the Nature Portfolio guidelines for submitting code & software for further information.

Data

Policy information about availability of data

All manuscripts must include a data availability statement. This statement should provide the following information, where applicable:

- Accession codes, unique identifiers, or web links for publicly available datasets
- A description of any restrictions on data availability
- For clinical datasets or third party data, please ensure that the statement adheres to our policy

Data availability.

All data that support the findings of this study are presented in this article, in Supplementary Information, and in the Source Data file. Additional information will be made available from the corresponding author upon reasonable request. Previously published structures used in this study (PDB IDs 6MM9 [https://www.rcsb.org/structure/6mm9] and 5WEO [https://www.rcsb.org/structure/5WEO]) can be accessed at the Protein Data Bank (https://www.rcsb.org/). Source data are provided with this paper.

Field-specific reporting

Please select the one below that is the best fit for your research. If you are not sure, read the appropriate sections before making your selection.

☒ Life sciences ☐ Behavioural & social sciences ☐ Ecological, evolutionary & environmental sciences

For a reference copy of the document with all sections, see nature.com/documents/nr-reporting-summary-flat.pdf

Life sciences study design

All studies must disclose on these points even when the disclosure is negative.

Sample size	Sample sizes were chose based on our results from relevant previous experiments in the articles Glasgow et al. 2017 (J Neurosci 37, 9686-9704) and Glasgow et al. 2018 (Neuropharmacol 137, 344-358).
Data exclusions	Data exclusion criteria were pre-established. MCI measurements were excluded if peak IControl2 and peak IControl1 differed by >20% (to avoid inaccurate current quantification due to response run-down or run-up). Cells were excluded from analysis if: peak NMDAR current was >2.5 nA or if series resistance was >20 Mohm (to minimize series resistance error); holding current was more negative than -200 pA (to avoid use of unhealthy cells); holding current fluctuations exceeded 100 pA during an experiment (to minimize inaccurate current quantification due to variation in holding current).
Replication	All electrophysiological experiments were repeated independently 3 to 12 times. Number of independent measurements (number of cells from which independent recordings were made) are indicated by n in figure legends, where n also is defined.
Randomization	Randomization is not applicable to experiments on cultured cells, receptor modeling, or chemical synthesis, the approaches used in this study, because samples were not separated into experimental groups.
Blinding	Blinding was not used for electrophysiological experiments because, for logistical reasons, the same experimenter performed transfection and recordings, precluding blinding. Identical use in all experiments of automated data acquisition and analysis procedures minimized the possibility that experimenter bias could influence results. Blinding to group allocation cannot be used for receptor modeling or chemical syntheses because no group allocation was performed.

Reporting for specific materials, systems and methods

We require information from authors about some types of materials, experimental systems and methods used in many studies. Here, indicate whether each material, system or method listed is relevant to your study. If you are not sure if a list item applies to your research, read the appropriate section before selecting a response.

Materials & experimental systems

n/a	Involved in the study
☒	☐ Antibodies
☐	☒ Eukaryotic cell lines
☒	☐ Palaeontology and archaeology
☐	☒ Animals and other organisms
☒	☐ Human research participants
☒	☐ Clinical data
☒	☐ Dual use research of concern

Methods

n/a	Involved in the study
☒	☐ ChIP-seq
☒	☐ Flow cytometry
☒	☐ MRI-based neuroimaging

Eukaryotic cell lines

Policy information about cell lines

Cell line source(s)	tsA201 obtained from The European Collection of Authenticated Cell Cultures
Authentication	The European Collection of Authenticated Cell Cultures authenticated tsA201 cells using Short Tandem Repeat (STR) profiling.
Mycoplasma contamination	Our cell lines tested negative for mycoplasma
Commonly misidentified lines (See ICLAC register)	No commonly misidentified cell lines were used

Animals and other organisms

Policy information about studies involving animals; ARRIVE guidelines recommended for reporting animal research

Laboratory animals	Pregnant adult female rats of the Sprague-Dawley strain were used on gestational day 16 for preparation of neuronal cultures from embryos.
Wild animals	The study did not involve wild animals
Field-collected samples	The study did not involve samples collected from the field
Ethics oversight	All procedures were approved by the Institutional Animal Care and Use Committee of the University of Pittsburgh

Note that full information on the approval of the study protocol must also be provided in the manuscript.
